# Nonlinear Attitude Control of a Spherical Underwater Vehicle †

**DOI:** 10.3390/s19061445

**Published:** 2019-03-24

**Authors:** Ramon A. Suarez Fernandez, E. Andres Parra R., Zorana Milosevic, Sergio Dominguez, Claudio Rossi

**Affiliations:** Centre for Automation and Robotics, Universidad Politecnica de Madrid, 28006 Madrid, Spain; ea.parra01@gmail.com (E.A.P.R.); zorana.milosevic@upm.es (Z.M.); sergio.dominguez@upm.es (S.D.); claudio.rossi@upm.es (C.R.)

**Keywords:** underwater robots, AUV, spherical robots, feedback linearization, mine exploration

## Abstract

In this work, we present the design, implementation, and testing of an attitude control system based on State Feedback Linearization (FL) of a prototype spherical underwater vehicle. The vehicle is characterized by a manifold design thruster configuration for both locomotion and maneuvering, as well as on a novel pendulum-based passive pitch control mechanism. First, the mechanical design and onboard electronics set up of the spherically shaped hull are introduced. Afterward, a high-fidelity dynamic model of the system is derived for a 6 degree-of-freedom (DOF) underwater vehicle, followed by several experiments that have been performed in a controlled environment to compare the performance of the proposed control method to that of a baseline Proportional-Integral-Derivative (PID) controller. Experimental results demonstrate that while both controllers were able to perform the specified maneuvers, the FL controller outperforms the PID in terms of precision and time response.

## 1. Introduction

Europe is coming under increased pressure due to its near-complete dependency on the import of mineral raw materials. At the same time, there are an estimated 30,000 inactive mining sites, a considerable number of which still contain raw materials, currently in critical demand [1]. Among these sought-after raw materials there are metallic and industrial minerals, construction materials, and base metals, such as cobalt, gallium, indium, and a range of rare earths necessary for IT appliances [2]. Therefore, there is increased interest for re-opening some of these abandoned mine sites.

Under normal operation conditions, surface and groundwaters filter into the mined tunnels and must be constantly removed to maintain a safe working environment. Once a mine is permanently closed, the dewatering systems cease to operate, and without the existence of any drainage, the tunnels become permanently submerged. Most of these mine sites, presently submerged, are more than a century old, and the information available regarding the structural layout of the tunnels is limited and imprecise, if not totally lacking. The network of tunnels inside a mine can be extremely complicated, so surveying and prospecting by conventional methods such as human divers, can result dangerous or even lethal in unknown deep mine conditions [1]. Therefore, the use of robotic platforms such as underwater vehicles to explore these sites and gather valuable geological and mineralogical information, can help reducing risks for humans and provide important information for determining whether re-opening a mine is plausible and economically feasible.

Underwater vehicles are an active area of research due to their potential applications in a variety of fields, such as maritime security, marine archaeology, search, and rescue. The most common applications of underwater robotics include ocean mining exploration [3], autonomous sea floor mapping [4,5], and data gathering [6,7,8]. It is worth noting that most of the research on underwater robots addresses open water environments, where the restrictions on shapes and sizes of the UVs are not strict. Therefore, most UVs adopt an elongated shape to minimize drag in surge (forward motion) which is the dominant motion in open water.

Nevertheless, for autonomous exploration and 3D mine mapping of flooded mines, these elongated shapes are not efficient. The underwater robot will have to navigate within complex structures of mine tunnels and galleries with dimensions in the range of 1.5 m × 3 m, with the oldest mines tunnel sections as small as 1.5 m × 1 m. As such, the robot must be sufficiently small and agile to navigate the mine corridors and tight turns of the mine system. Furthermore, mine tunnels may contain objects or debris left after closure, which could obstruct paths or become entangled in the AUVs propellers and permanently disable the robot which implies certain design requirements aimed at limiting protruding elements to avoid becoming snared. By adopting a less common streamlined spherical shape design, attitude motions can be made in-place, minimizing the number of moving parts and protruding elements while obtaining high stability and flexibility along with a zero degree turn radius for high maneuverability and lower drag forces.

Although spherical hull designs for underwater vehicles are not commonly found in recent literature, because of the preferred bullet-shape hull, such as the MAYA AUV [9], developed by the National Institute of Oceanography in India, several works can be found which took advantage of this particular shape. The UV design patented by the authors in [10] is one of the first spherical underwater robots where the spherical shape of the prototype is used to obtain lower drag forces during operation. It is composed of four small engines that help stabilize and maneuver the vehicle, and a single engine that is in the center of the sphere for propulsion of the UV. The University of Hawaii developed ODIN-III [11], a prototype robot with a hollow metal sphere housing of 0.315 m in radius and a propulsion system that consisted of 8 screw propellers fixed outside the hull. This platform has been used to test adaptive control methods together with disturbance observers [12]. A micro AUV of 0.075 m in radius and 6 propellers around the hull was developed by the authors of [13,14] for monitoring sub-surface cluttered environments as in nuclear storage ponds.

**Figure 1:**
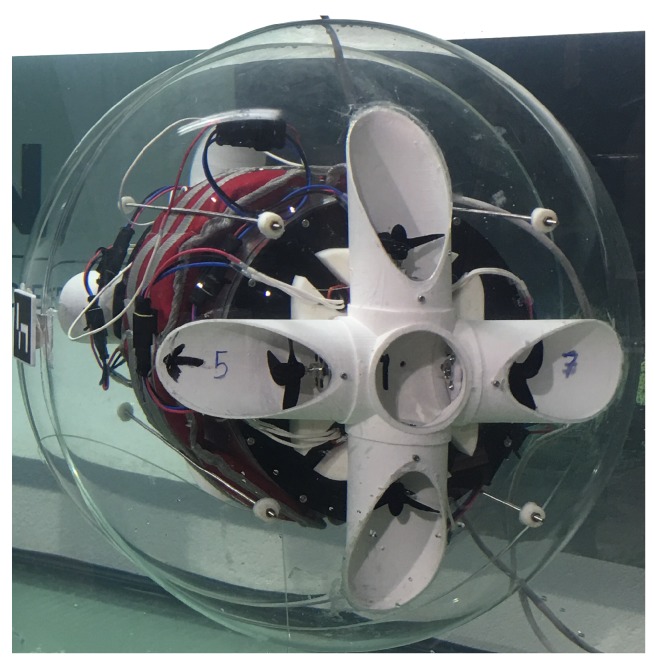
Spherical underwater vehicle prototype during pool tests.

Screw propeller thrusters have been generally used for propulsion of underwater vehicles, nevertheless, other types of propulsion methods such as water-jet thrusters have been designed and tested for spherical UV. The robot developed in [15] is a prototype that has a spherical hull with a diameter of 0.40 m and uses 4 vector water-jets as propulsion to maneuver, which is based on the design patented in [16]. Although this UV has 4 vector water-jet inside, it is only capable of performing motions in 3 DOF [17,18,19]. The authors in [20] make use of the spherical UV SUR-II, which is equipped with water-jet thrusters as propulsion, to design and develop attitude stabilization methods as well as buoyancy control with a variable ballast tank. Whereas, spherical AUVs with reaction wheel internal actuators have been designed and developed in [21,22]. One key disadvantage of these UV designs is the presence of external propulsion systems which could become entangled with objects encountered during operation. Taking into account the benefits and drawbacks of these systems, the UV design in this work shown in Figure 1, has a manifold thruster configuration where the propulsion elements are embedded into the spherical hull to avoid foreign objects from damaging the propellers and effectively eliminating the possibility of ensnarement.

The work presented in this paper describes a scaled prototype UV, realized to study, develop, and compare control strategies, to be later deployed, with suitable adaptations, in the actual robotic explorer for the UNEXMIN project, hereby named UX-1. Here, we focus on three aspects: the mechanical and electrical design of the spherical UV prototype, the derivation of the equations of motion that describe the dynamics of the system and the implementation of control methods for performance analysis in real underwater experiments. The rest of this paper is organized as follows: Section 2 introduces the mechanical and electronic design of the UV, while in Section 3 the 6 DOF equations of motion for an UV are derived. Section 4 explains the control system implemented on the UV, while the underwater experimental tests and discussions are reported in Section 5. Finally, conclusions and future works are presented in Section 6.

## 2. Underwater Vehicle Design

The mechanical design of the scaled UV prototype (Figure 1) is focused on complying to the expected requirements of the real UNEXMIN UX-1 robot being developed and deployed in real mine scenarios for the task of flooded tunnel navigation. For efficiency in design, development, and testing of the UV prototype, low-cost COTS mechanical and electronic components were used, when available, so that any component damaged during testing could be easily replaced. The final prototype’s mechanical and electronic design has been thoroughly explained in [23] and can be divided into three main components, which are: external hull, manifold system for propulsion and a pendulum mechanism for pitch movement.

### 2.1. External Hull

**Figure 2:**
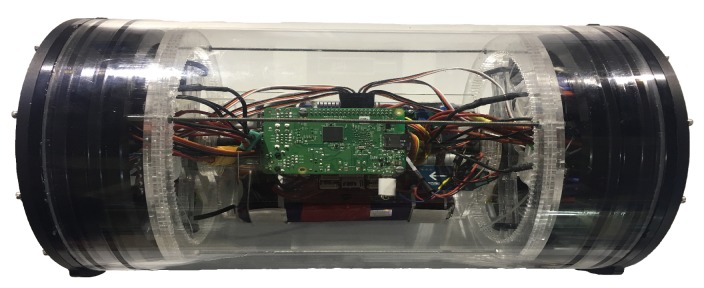
Watertight Enclosure used for electronic devices in the prototype UV.

The design requirements for the external hull are based on the smallest area where the robot will have to navigate. In most flooded mine tunnels this area will be found in the staircases interconnecting tunnels and galleries. The size restriction was fixed at a maximum diameter of 60 cm. Thus, the external hull of the UV prototype was chosen as two acrylic hemispheres of 50 cm in diameter integrated with the manifold system and rigidly attached to a watertight enclosure for electronics shown in Figure 2.

### 2.2. Manifold Propulsion System

**Figure 3:**
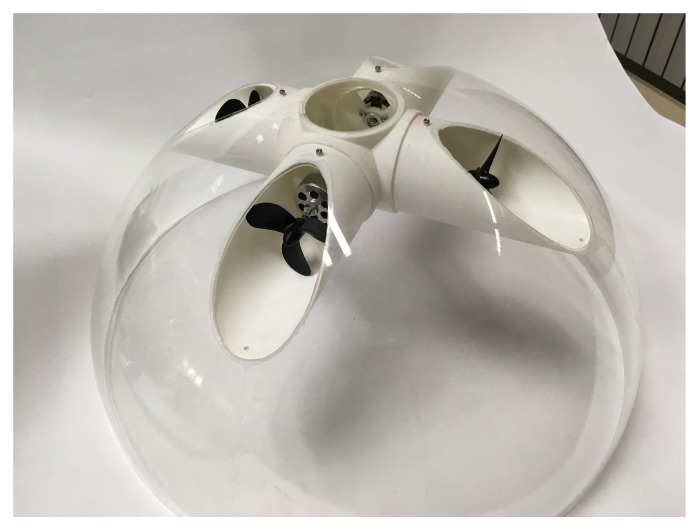
Final placement of the manifolds in a hemisphere of the prototype UV.

In the scaled UV prototype, the manifold component, shown in Figure 3, is assembled from 3D-printed ABS and incorporated into each lateral hemisphere (port and starboard) of the streamlined spherical shape of the hull, along with two counter rotating vertical and horizontal motors.

**Figure 4:**
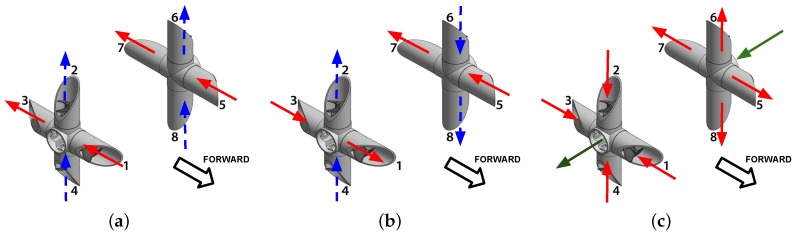
Thruster direction for UV movement. (**a**) Surge (red) and Heave (blue) motions; (**b**) Yaw (red) and Roll (blue) motions (**c**) Sway motion (red) and flow force direction (green).

Given the implicit symmetry of the manifold design and the properties of fluid dynamics, a translation in any direction is possible without changing the heading of the UV [24], nevertheless, pitch rotational motion is not possible. Figure 4 shows the id of each actuator and the direction of the thrust for each motion possible which are outlined in Table 1.

As can be seen in Figure 4a, Surge (red) and Heave (blue) motions are accomplished by applying equal force to port and starboard thrusters, while Yaw (red) and Roll (blue) motions (Figure 4b) are obtained by differential control inputs. Finally, Sway motion, shown in Figure 4c, is performed by rotating the thrusters on each manifold in the same direction (outward/inward), thus directing the flow of water through the center orifice of the manifold.

### 2.3. Pendulum Mechanism

In the foreseen exploration missions, navigation will be mainly performed with the front of the robot facing the direction of movement, since most of the sensors for 3D mapping and localization on the UX-1 are in the front of the robot. Thus, if it is expected that vertical or steep tunnels are to be explored, and a given pitch angle must be maintained for relatively long periods of time, the sustained use of propellers would consume significant battery power. Hence, passive pitch stabilization is achieved with the use of an internal pendulum mechanism that rotates a mass suspended below the CB around the $y_{b}$ axis of the vehicle.

**Figure 5:**
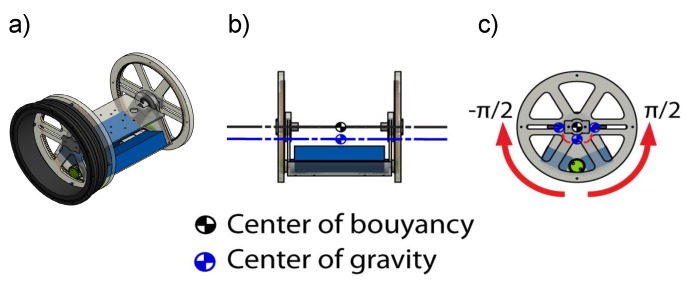
(**a**) Pendulum mechanism assembled in flange of watertight container (**b**) front view and (**c**) side view of pitch motion acquired by pendulum shift.

To the best of our knowledge, this is the first time this design is used for pitch control of a spherical underwater vehicle. As seen in Figure 5 the design consists of two fixed bases and an electronics tray rigidly mounted to the watertight enclosure’s flange seal (Figure 2), and a mobile base that is moved along the rotation axis by two DC motors and a rack-and-pinion gear design.

## 3. Equations of Motion

**Figure 6:**
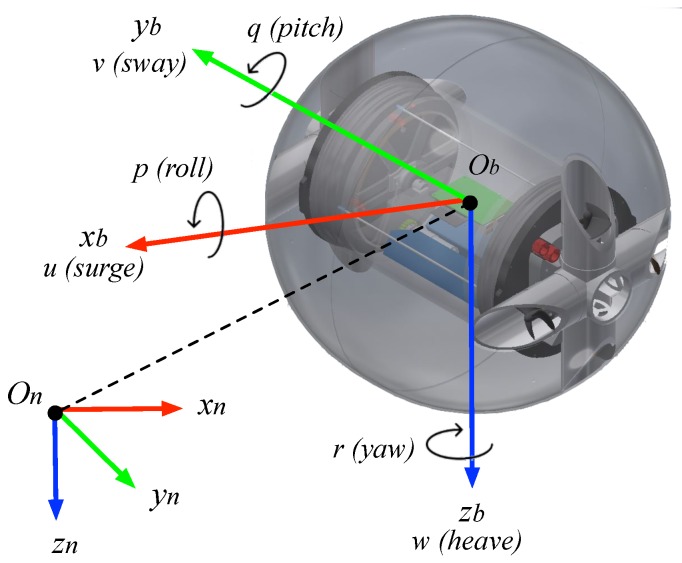
Schematics of the developed underwater vehicle with the frames of reference used in the equations of motion.

The 6 DOF equations of motion for an UV are derived according to the SNAME nomenclature [25]. Given that for the experiments performed in this work the UV is not meant to be actuated in all degrees of freedom, reduced order models are presented for longitudinal motion (Section 3.2.1) and lateral motion (Section 3.2.2), such as in [23]. As can be seen in Figure 6, we make use of an inertial frame in NED configuration {n}, with orthonormal basis $\{x_{n}$, $y_{n}$, $z_{n}\}$ and origin $o_{n}$ represented in world coordinates, as well as the body-fixed reference frame {b} with orthonormal basis $\{x_{b}$, $y_{b}$, $z_{b}\}$ and origin $o_{b}$ also in world coordinates.

### 3.1. Nonlinear 6 DOF Model

Since the equations of motion of an UV moving at high speeds are highly nonlinear and coupled, in this work the vehicle is restricted to perform low speed maneuvers in translation motions, namely, the speed of the robot will be limited to a maximum of 0.25 m/s. Furthermore, this value has been chosen as the maximum normal operating speed for the UX-1 robot during future exploration missions. We consider the nonlinear equations of motion developed in [23,26] for an UV:

(1) $$\dot{\eta }=J_{\Theta }\left(\eta \right)\nu$$

(2) $$M\dot{\nu }+C\left(\nu \right)\nu +D\left(\nu \right)\nu +g\left(\eta \right)=\tau$$

with:

(3) $$\eta =\left[\begin{array}{c} P_{b/n}^{n}\\ \Theta_{nb} \end{array}\right]={\left[x,y,z,\phi ,\theta ,\psi \right]}^{⊤}$$

(4) $$J_{\Theta }\left(\eta \right)=\left[\begin{array}{cc} R_{b}^{n}\left(\Theta_{nb}\right) & 0_{3\times 3}\\ 0_{3\times 3} & T_{\Theta }\left(\Theta_{nb}\right) \end{array}\right]$$

(5) $$\nu =\left[\begin{array}{c} v_{b/n}^{b}\\ \omega_{b/n}^{b} \end{array}\right]={\left[u,v,w,p,q,r\right]}^{⊤}$$

(6) $$\tau =\left[\begin{array}{c} f_{b}^{b}\\ m_{b}^{b} \end{array}\right]={\left[X,Y,Z,K,M,N\right]}^{⊤}.$$

#### 3.1.1. Nomenclature

The dynamic equations in (2) are composed of the system inertia matrix ***M***, the Coriolis and Centripetal term matrix ***C***(***ν***), the hydrodynamic damping matrix ***D***(***ν***), and the vector of hydrostatic forces and moments *g*(***η***). In (3), we denote the position ($P_{b/n}^{n}$) and orientation (**Θ**_*nb*_) vector in the NED coordinate system as ***η*** ∈ ℝ^3^ × *S*^3^, where $P_{b/n}^{n}$ ∈ ℝ^3^ is defined as the distance of the point *o_b_* with respect to {*n*} expressed in {*n*} and **Θ**_*nb*_ ∈ *S*^3^ is a vector of Euler angles between {*n*} and {*b*}. The linear ($v_{b/n}^{b}$) and angular ($\omega_{b/n}^{b}$) velocity vectors in the body-fixed reference frame are denoted as ***ν*** ∈ ℝ^6^ in (5). ***J***_**Θ**_(***η***) is a 6 × 6 transformation matrix formed by a rotation matrix $R_{b}^{n}$(**Θ**_*nb*_), for transforming $v_{b/n}^{b}$ in {*b*} to {*n*}, and a transformation matrix ***T***_Θ_(**Θ**_*nb*_) to relate $\omega_{b/n}^{b}$ in {*b*} to the Euler rate vector ${\dot{\Theta }}_{nb}$. Lastly, ***τ*** ∈ ℝ^6^ is used to describe the forces ($f_{b}^{b}$) and moments ($m_{b}^{b}$) acting on the vehicle in the body-fixed reference frame.

#### 3.1.2. System Inertia Matrix

The system inertia matrix is a positive semi-definite matrix composed of the rigid-body inertia matrix $M_{RB}$ and the added mass inertia matrix $M_{A}$, composed as follows:

(7) $$M=M_{RB}+M_{A},\;M=M^{⊤}>0$$

with,

(8) $$\begin{array}{c} M_{RB}=\left[\begin{array}{cccccc} m & 0 & 0 & 0 & mz_{g} & -my_{g}\\ 0 & m & 0 & -mz_{g} & 0 & mx_{g}\\ 0 & 0 & m & my_{g} & -mx_{g} & 0\\ 0 & -mz_{g} & my_{g} & I_{xx} & -I_{xy} & -I_{xz}\\ mz_{g} & 0 & -mx_{g} & -I_{yx} & I_{yy} & -I_{yz}\\ -my_{g} & mx_{g} & 0 & -I_{zx} & -I_{zy} & I_{zz} \end{array}\right] \end{array}$$

which satisfies, $M_{RB}=M_{RB}^{⊤}>0$ and ${\dot{M}}_{RB}=0_{6\times 6}$. In (8), *m* is the mass of the vehicle, $\left\{I_{xx},I_{yy},I_{zz}\right\}$ are the moments of inertia about {b} axes, $\left\{I_{xy}=I_{yx},I_{xz}=I_{zx},I_{yz}=I_{zy}\right\}$ are the products of inertia, and $\left\{x_{g},y_{g},z_{g}\right\}$ are the distances from the geometrical center of the vehicle to the CG. In the case of a spherical UV with symmetry in the xz, yz and xy planes ($x_{g}=y_{g}=z_{g}\approx 0$), the rigid-body matrix can be approximated by:

(9) $$M_{RB}=M_{RB}^{⊤}\approx diag\left\{m,m,m,I_{xx},I_{yy},I_{zz}\right\}.$$

The 6 × 6 inertia matrix of added mass terms $M_{A}$ describes the mass that the UV must displace while moving, and the inertia due to the displaced mass while rotating [27]. Given that the UV is restricted to low speed maneuvers and the fact that the vehicle design in this work has three planes of symmetry, the contribution of the off-diagonal elements in $M_{A}$ can be neglected. Hence,

(10) $$M_{A}=M_{A}^{⊤}\approx -diag\left\{X_{\dot{u}},Y_{\dot{v}},Z_{\dot{w}},K_{\dot{p}},M_{\dot{q}},N_{\dot{r}}\right\}.$$

The added mass matrix elements $X_{\dot{u}}$, $Y_{\dot{v}}$, $Z_{\dot{w}}$, $K_{\dot{p}}$, $M_{\dot{q}}$ and $N_{\dot{r}}$ are the hydrodynamic derivatives of added mass forces as explained in [27]. These forces can be described by an axial component ($X_{\dot{u}}$, $Y_{\dot{v}}$, $Z_{\dot{w}}$) and a rolling component ($K_{\dot{p}}$, $M_{\dot{q}}$, $N_{\dot{r}}$). Assuming that the shape of the UV can be approximated by an ellipsoid, the axial component of the hydrodynamic added mass force *X* along the *x* axis due to an acceleration $\dot{u}$ in the *x* direction is computed as:

(11) $$X_{\dot{u}}=\frac{4\;\beta \;\rho \;\pi }{3}{\left(\frac{d}{2}\right)}^{3}$$

where ρ is the water density, *d* is the diameter of the vehicle, and β is a coefficient based on the ratio between the vehicle length and diameter. Similar considerations hold for the axial components of the hydrodynamic added mass force, *Y* and *Z*. Furthermore, the mass displaced while rotating the spherical UV is negligible, consequently the last three elements of (10) can be approximated to zero.

#### 3.1.3. Coriolis-Centripetal Matrix

The Coriolis and Centripetal term matrix (12) is caused by the rotation of the body with respect to the inertial reference frame.

(12) $$C\left(\nu \right)=C_{RB}\left(\nu \right)+C_{A}\left(\nu \right)$$

$C_{RB}\left(\nu \right)$ and $C_{A}\left(\nu \right)$ are the rigid-body and hydrodynamic Coriolis and Centripetal matrices, respectively. The theory and proofs of these matrices can be found thoroughly explained in literature [26]. It is generally good practice to design the system with the CG located lower than the CB to stabilize the system. For the spherical UV if the CG and the CB are located vertically on the *z* axis, that is, $x_{b}=x_{g}\approx 0$ and $y_{b}=y_{g}\approx 0$ and $I_{xy}$, $I_{xz}$, $I_{yz}$≈ 0, the rigid-body Coriolis and Centripetal matrix is defined as:

(13) $$\begin{array}{c} C_{RB}\left(\nu \right)=\left[\begin{array}{cccccc} 0 & 0 & 0 & mz_{g}r & mw & -mv\\ 0 & 0 & 0 & -mw & mz_{g}r & mu\\ 0 & 0 & 0 & -m\alpha_{1} & -m\alpha_{2} & 0\\ -mz_{g}r & mw & m\alpha_{1} & 0 & I_{zz}r & -I_{yy}q\\ -mw & -mz_{g}r & m\alpha_{2} & -I_{zz}r & 0 & I_{xx}p\\ mv & -mu & 0 & I_{yy}q & -I_{xx}p & 0 \end{array}\right] \end{array}$$

with

(14) $$\alpha_{1}=\left(z_{g}p-v\right),\;\alpha_{2}=\left(z_{g}q+u\right).$$

The nonlinear hydrodynamic Coriolis and Centripetal matrix $C_{A}\left(\nu \right)$ due to a rotation of the body reference frame about the inertial frame can be derived using an energy formulation based on the added mass matrix $M_{A}$ [27].

(15) $$\begin{array}{c} C_{A}\left(\nu \right)=\left[\begin{array}{cccccc} 0 & 0 & 0 & 0 & -Z_{\dot{w}}w & Y_{\dot{v}}v\\ 0 & 0 & 0 & Z_{\dot{w}}w & 0 & -X_{\dot{u}}u\\ 0 & 0 & 0 & -Y_{\dot{v}}v & X_{\dot{u}}u & 0\\ 0 & -Z_{\dot{w}}w & Y_{\dot{v}}v & 0 & -N_{\dot{r}}r & M_{\dot{q}}q\\ Z_{\dot{w}}w & 0 & -X_{\dot{u}}u & N_{\dot{r}}r & 0 & -K_{\dot{p}}p\\ -Y_{\dot{v}}v & X_{\dot{u}}u & 0 & -M_{\dot{q}}q & K_{\dot{p}}p & 0 \end{array}\right] \end{array}$$

#### 3.1.4. Damping Matrix

The total hydrodynamic damping matrix D(ν) is the sum of the linear term D and the nonlinear term $D_{n}\left(\nu \right)$ such that

(16) $$D\left(\nu \right)=D+D_{n}\left(\nu \right)$$

If the UV is maneuvering at low speeds, the motions can be considered uncoupled, thus D(ν) could be assumed mathematically as [26]:

(17) $$\begin{array}{cc} D\left(\nu \right)=-diag\left\{X_{u},Y_{v},Z_{w},K_{p},M_{q},N_{r}\right\} & \\ & \;-diag\{X_{|u|u}|u|,Y_{|v|v}|v|,Z_{|w|w}|w|,K_{|p|p}|p|,M_{|q|q}|q|,N_{|r|r}|r|\} \end{array}$$

where $\left\{X_{u},Y_{v},Z_{w},K_{p},M_{q},N_{r}\right\}$ are the linear damping coefficients of D and $\left\{X_{|u|u},Y_{|v|v},Z_{|w|w},K_{|p|p},M_{|q|q},N_{|r|r}\right\}$ are the axial and rolling drag parameters of $D_{n}\left(\nu \right)$. Definitions and theory can be found in [28].

#### 3.1.5. Vector of Gravitational Forces and Moments

Conforming to the SNAME nomenclature, the weight and buoyancy of a submerged vehicle is given by

(18) W=mg,B=ρg∇

where *g* is the acceleration of gravity in NED, ∇ is the volume of fluid displaced by the vehicle and ρ is the density of water. As in Section 3.1.3, if $x_{b}=x_{g}\approx 0$ and $y_{b}=y_{g}\approx 0$, the Euler angle representation of hydrostatic forces and moments for the gravitational and buoyant forces acting on the vehicle is given by [27]:

(19) $$g\left(\nu \right)=\left[\begin{array}{c} (W-B)\sin\theta \\ -(W-B)\cos\theta \sin\phi \\ -(W-B)\cos\theta \cos\phi \\ (z_{g}W-z_{b}B)\cos\theta \sin\phi \\ (z_{g}W-z_{b}B)\sin\theta \\ 0 \end{array}\right].$$

As can be seen in (19) if the UV is close to neutrally buoyant (W=B), the gravitational forces only affect the rotations in the *x* and *y* axes.

### 3.2. Reduced Order Models

In certain cases, the nonlinear equations of motion presented in Section 3.1 can be divided into two slightly interacting subsystems. In the longitudinal subsystem the states are chosen to be {u,w,q,θ} while the remaining states (e.g., {v,p,r,ϕ,ψ}) are those of the lateral subsystem. From the diagonal structure of (7)–(10), it can be observed in (20) that the two subsystems are clearly decoupled.

(20) $$M_{long}=\left[\begin{array}{ccc} M_{11} & 0 & 0\\ 0 & M_{33} & 0\\ 0 & 0 & M_{55} \end{array}\right]M_{lat}=\left[\begin{array}{ccc} M_{22} & 0 & 0\\ 0 & M_{44} & 0\\ 0 & 0 & M_{66} \end{array}\right]$$

#### 3.2.1. Longitudinal Model

In the longitudinal model, the states of the lateral subsystem are assumed to be small (v=p=r=ϕ=ψ≈0). Therefore, the longitudinal kinematics for *u*, *w* and θ are [27],

(21) $$\left[\begin{array}{c} \dot{x}\\ \dot{z}\\ \dot{\theta } \end{array}\right]=\left[\begin{array}{cc} \sin\theta & 0\\ \cos\theta & 0\\ 0 & 1 \end{array}\right]\left[\begin{array}{c} w\\ q \end{array}\right]+\left[\begin{array}{c} \cos\theta \\ -\sin\theta \\ 0 \end{array}\right]u.$$

To reduce the complexity of the system, the higher order damping terms $D_{n}\left(\nu \right)$ in (16) are neglected. Nevertheless, the Coriolis-Centripetal matrix (12) is modelled assuming that v,w,p,q and *r* are small and u>>0. Thus, using (13)–(15),

(22) $$C_{RB}\left(\nu \right)\nu \approx \left[\begin{array}{ccc} 0 & 0 & 0\\ 0 & 0 & -mu\\ 0 & 0 & 0 \end{array}\right]\left[\begin{array}{c} u\\ w\\ q \end{array}\right]$$

(23) $$C_{A}\left(\nu \right)\nu \approx \left[\begin{array}{ccc} 0 & 0 & 0\\ 0 & 0 & X_{\dot{u}}u\\ 0 & (Z_{\dot{w}}-X_{\dot{u}})u & 0 \end{array}\right]\left[\begin{array}{c} u\\ w\\ q \end{array}\right].$$

Therefore, according to (2), (7), (17), (19), the dynamics become

(24) $$\begin{array}{c} \left[\begin{array}{ccc} m-X_{\dot{u}} & 0 & 0\\ 0 & m-Z_{\dot{w}} & 0\\ 0 & 0 & I_{y}-M_{\dot{q}} \end{array}\right]\left[\begin{array}{c} \dot{u}\\ \dot{w}\\ \dot{q} \end{array}\right]+\left[\begin{array}{ccc} -X_{u} & 0 & 0\\ 0 & -Z_{w} & 0\\ 0 & 0 & -M_{q} \end{array}\right]\left[\begin{array}{c} u\\ w\\ q \end{array}\right]\\ \;+\left[\begin{array}{ccc} 0 & 0 & 0\\ 0 & 0 & \gamma_{1}u\\ 0 & (Z_{\dot{w}}-X_{\dot{u}})u & 0 \end{array}\right]\left[\begin{array}{c} u\\ w\\ q \end{array}\right]+\left[\begin{array}{c} (W-B)\sin\theta \\ -(W-B)\cos\theta \cos\phi \\ (z_{g}W-z_{b}B)\sin\theta \end{array}\right]=\left[\begin{array}{c} \tau_{1}\\ \tau_{3}\\ \tau_{5} \end{array}\right] \end{array}$$

for $\gamma_{1}=\left(X_{\dot{u}}-m\right)$. As mentioned before, the longitudinal motions for Surge (*u*) and Heave (*w*) are controlled using the thruster configuration with the manifolds while the pitch (ϕ) motion is controlled using the internal pendulum mechanism (Section 2.3).

#### 3.2.2. Lateral Model

For the lateral model, the longitudinal subsystem states {u,w,q and θ} and the roll angle (ϕ) are assumed to be small. The lateral kinematics result in

(25) $$\left[\begin{array}{c} \dot{y}\\ \dot{\phi }\\ \dot{\psi } \end{array}\right]=\left[\begin{array}{ccc} \cos\psi & 0 & 0\\ 0 & 1 & 0\\ 0 & 0 & 1 \end{array}\right]\left[\begin{array}{c} v\\ p\\ r \end{array}\right].$$

As in the previous Section 3.2.1, the higher order terms in (16) are neglected and the Coriolis terms in (12) are modelled assuming that v,w,p,q, and *r* are negligible. Hence from (13)–(15),

(26) $$C_{RB}\left(\nu \right)\nu \approx \left[\begin{array}{ccc} 0 & 0 & mu\\ 0 & 0 & -mz_{g}u\\ 0 & 0 & 0 \end{array}\right]\left[\begin{array}{c} v\\ p\\ r \end{array}\right]$$

(27) $$C_{A}\left(\nu \right)\nu \approx \left[\begin{array}{ccc} 0 & 0 & -X_{\dot{u}}u\\ 0 & 0 & 0\\ (X_{\dot{u}}-Y_{\dot{v}})u & 0 & 0 \end{array}\right]\left[\begin{array}{c} v\\ p\\ r \end{array}\right]$$

Therefore, the dynamics of the lateral subsystem become

(28) $$\begin{array}{c} \left[\begin{array}{ccc} m-Y_{\dot{v}} & 0 & 0\\ 0 & I_{x}-K_{\dot{p}} & 0\\ 0 & 0 & I_{z}-N_{\dot{r}} \end{array}\right]\left[\begin{array}{c} \dot{v}\\ \dot{p}\\ \dot{r} \end{array}\right]+\left[\begin{array}{ccc} -Y_{v} & 0 & 0\\ 0 & -K_{p} & 0\\ 0 & 0 & -N_{r} \end{array}\right]\left[\begin{array}{c} v\\ p\\ r \end{array}\right]\\ \;+\left[\begin{array}{ccc} 0 & 0 & \gamma_{2}u\\ 0 & 0 & -mz_{g}u\\ \gamma_{4}u & 0 & 0 \end{array}\right]\left[\begin{array}{c} v\\ p\\ r \end{array}\right]+\left[\begin{array}{c} -(W-B)\cos\theta \sin\phi \\ \gamma_{3}\cos\theta \sin\phi \\ 0 \end{array}\right]=\left[\begin{array}{c} \tau_{2}\\ \tau_{4}\\ \tau_{6} \end{array}\right] \end{array}$$

where, $\gamma_{2}=\left(m-X_{\dot{u}}\right);\gamma_{3}=\left(z_{g}W-z_{g}B\right);\gamma_{4}=\left(X_{\dot{u}}-Y_{\dot{v}}\right)$.

## 4. Control System Design

The spherical UV prototype was developed to test advanced control algorithms before implementing them in the UX-1 system during future mine site tests. In this work a State Feedback Linearization (FL) control algorithm is implemented and compared to a baseline controller developed and tested previously in [23].

### 4.1. State Feedback Linearization Controller

**Figure 7:**
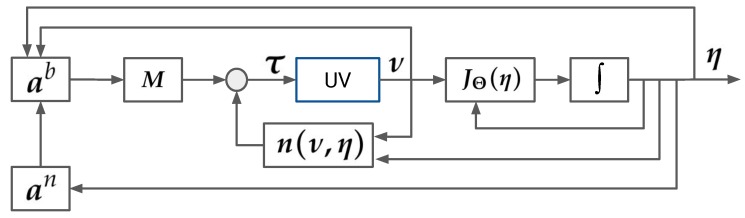
Feedback Linearization control block diagram with nonlinear decoupling in NED frame and transformation to the Body frame.

The State FL control method, shown in Figure 7, has been implemented and validated in a number works in the literature. In [29], the authors developed a FL controller for an UV with a linear Proportional-Derivative compensator for stabilization. The authors in [30,31] implemented heading controllers using a closed-loop gain shaping algorithm and a fault tolerant heading control system using the method of input-output FL, respectively. The main principle with FL is the transformation of the nonlinear dynamics of the system into a linear system:

(29) $$\ddot{\eta }=a^{n}$$

(30) $$\dot{\nu }=a^{b}$$

to which traditional control methods (e.g., Linear Quadratic Regulator, pole-placement) can be applied [27]. In (29), $a^{n}$ is interpreted as the commanded acceleration in the NED frame and in (30), $a^{b}$ is interpreted as the body-fixed commanded acceleration vector. We consider the nonlinear kinematic and kinetic equations from Section 3.1 in the form

(31) $$\dot{\eta }=J_{\Theta }\left(\eta \right)\nu$$

(32) $$M\dot{\nu }+n\left(\nu ,\eta \right)=\tau$$

where η and ν assumed to be measurable and n is the nonlinear vector of model parameters such that

(33) n(ν,η)=C(ν)ν+D(ν)ν+g(η)

The FL algorithm considers a complete knowledge of the nonlinear vector of model parameters in (33). As seen in Section 3, these parameters are generally hard to estimate and often inaccurate. This is a critical step since parametric inaccuracy can destabilize a feedback control system. Often better results are obtained when uncertain terms are chosen to be zero in the controller [27]. Nevertheless, by understanding the physical properties of the model (e.g., geometry, symmetry), it is possible to know which terms in the model can be omitted when deriving a model-based nonlinear controller. The control law is selected such that the nonlinearities of the system dynamics can be canceled out

(34) $$\tau =Ma^{b}+n\left(\nu ,\eta \right)$$

Differentiating (31) with respect to time and applying the control law (34) to the UV dynamics in (32) yields

(35) $$M\left(\dot{\nu }-a^{b}\right)=MJ_{\Theta }^{-1}\left(\eta \right)\left[\ddot{\eta }-{\dot{J}}_{\Theta }\left(\eta \right)\nu -J_{\Theta }\left(\eta \right)a^{b}\right]=0$$

From (35) it can be seen that by choosing

(36) $$a^{n}={\dot{J}}_{\Theta }\left(\eta \right)\nu +J_{\Theta }\left(\eta \right)a^{b}$$

results in the linear decoupled system $M^{*}\left(\ddot{\eta }-a^{n}\right)=0$ where $M^{*}=J_{\Theta }^{-}\left(\eta \right)MJ_{\Theta }^{-1}\left(\eta \right)>0$. From (36) it can be concluded that the commanded acceleration in the body-fixed frame $a^{b}$ can be calculated by

(37) $$a^{b}=J_{\Theta }^{-1}\left(\eta \right)\left[a^{n}-{\dot{J}}_{\Theta }\left(\eta \right)\nu \right]$$

(38) $$a^{n}={\ddot{\eta }}_{d}-K_{d}\dot{\tilde{\eta }}-K_{p}\tilde{\eta }-K_{i}\int_{0}^{t}\tilde{\eta }\left(\tau \right)d\tau$$

where $\eta_{d}$ is the desired position and orientation vector in NED, $\tilde{\eta }=\eta -\eta_{d}$ the position and orientation tracking error and $K_{p}$, $K_{i}$ and $K_{d}$ are positive definite matrices of the controller gains which can be chosen such that the error dynamics

(39) $${\ddot{\eta }}_{d}+K_{d}\dot{\tilde{\eta }}+K_{p}\tilde{\eta }+K_{i}\int_{0}^{t}\tilde{\eta }\left(\tau \right)d\tau =0$$

is globally exponentially stable.

### 4.2. Pole-Placement

Let Λ>0 be defined as a diagonal design matrix

(40) $$\Lambda =diag\{\lambda_{1},\lambda_{2},\ldots ,\lambda_{n}\}$$

used to specify the control bandwidth, $\eta_{d}$ the desired position and orientation vector in NED, and $\tilde{\eta }=\eta -\eta_{d}$ the position and orientation tracking error. Then the commanded acceleration in the NED frame can be chosen as a PID control law with acceleration feedforward,

(41) $$a^{n}={\ddot{\eta }}_{d}-K_{d}\dot{\tilde{\eta }}-K_{p}\tilde{\eta }-K_{i}\int_{0}^{t}\tilde{\eta }\left(\tau \right)d\tau$$

where $K_{p}$, $K_{i}$ and $K_{d}$ are positive definite matrices of the controller gains which can be chosen such that the error dynamics

(42) $${\ddot{\eta }}_{d}+K_{d}\dot{\tilde{\eta }}+K_{p}\tilde{\eta }+K_{i}\int_{0}^{t}\tilde{\eta }\left(\tau \right)d\tau =0$$

is globally exponentially stable. A simple pole-placement algorithm for PID control can be chosen as:

(43) $${\left(s+\lambda_{i}\right)}^{3}\int_{0}^{t}\tilde{\eta }\left(\tau \right)d\tau =0\;\left(i=1,2,\ldots ,n\right)$$

where the poles for each DOF are in $s=-\lambda_{i}\;\left(i=1,2,\ldots ,n\right)$, which yields

(44) $$\begin{array}{cc} K_{p} & =3\Lambda^{2}=diag\left\{3\lambda_{1}^{2},3\lambda_{2}^{2},\ldots ,3\lambda_{n}^{2}\right\}\\ K_{i} & =\Lambda^{3}=diag\left\{\lambda_{1}^{3},\lambda_{2}^{3},\ldots ,\lambda_{n}^{3}\right\}\\ K_{p} & =3\Lambda =diag\{3\lambda_{1},3\lambda_{2},\ldots ,3\lambda_{n}\}. \end{array}$$

## 5. Experiments and Discussion

The experimental tests were designed to compare the performance of the FL controller, presented in Section 4.1, with the PID controller previously implemented and tested in [23] for a regulation problem scenario [32]. Thus, by comparing the ability of the UV to reach and maintain a desired position for longitudinal motions and heading control, an appropriate controller can be chosen for implementation in the real UX-1 robot. This type of maneuver is key for the overall purpose of the UNEXMIN project, which involves data gathering from sensitive scientific instruments (e.g., multi-spectral cameras) and a 3D mapping of the environment at specific locations inside the flooded mine tunnels which must not be corrupted by unstable control.

### 5.1. Experimental Setup

**Figure 8:**
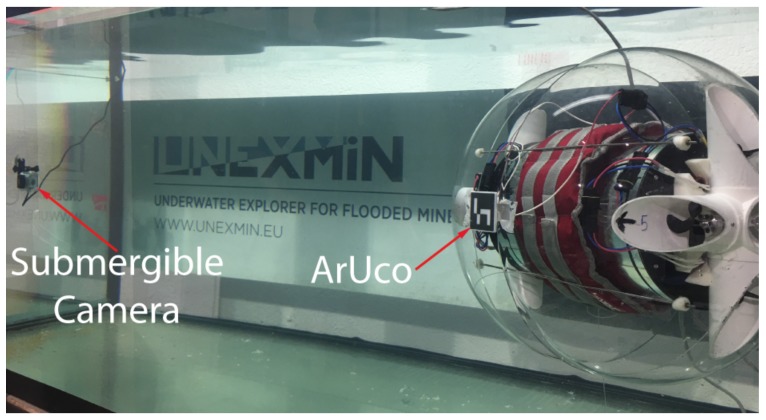
Underwater tests experimental setup. A submergible camera was installed inside the tank to estimate the position of the UV.

A 2 m × 1 m × 1 m water tank was used as the controlled environment to evaluate the position hold performance of the UV in several underwater tests. The feedback for the control algorithms was obtained using various methods. Surge (*x*) positions and Yaw (ψ) angles were acquired based on the works presented by [33,34], where the fiducial marker ArUco [35] was placed on the UV to estimate the attitude of the vehicle regarding a submersible camera installed inside the tank, as shown in Figure 8; Heave (*z*) depth measurements were read from the onboard pressure sensor and the Pitch (θ) feedback was obtained from the onboard gyroscope measurements in the Pixhawk Autopilot.

All hardware interfaces have been implemented in Python or c++ in Ubuntu 16.04 and data communications are handled using the ROS (https://www.ros.org/) middleware standard messages. Given the lack of available small low frequency wireless communication modules, a tether cable was used for real time data visualization, acquisition, and tuning. As can be seen in (18) the buoyancy of the vehicle depends on the volume of fluid displaced by the body. In the case of the UV in this work, the buoyancy is much larger than the weight of the vehicle and thus dead weights must be added in order to obtain approximate neutral buoyancy; however, in practice, it is a common practice to trim the weight of the robot as to have slightly positive buoyancy so that the UV in case of failure does not sink to the bottom. Lead weights were added in the flanges inside the watertight enclosure in addition to a sand bag weight around the watertight container for a total of 6kg in dead weight.

### 5.2. Underwater Experiments

In this work, the longitudinal motions (Surge (*x*), Heave (*z*) and Pitch (θ)) as well as the Yaw (ψ) of the UV were tested in underwater experiments of position hold performance with the FL controller. Given that the Roll (ϕ) movement will not be used in data collection during the missions and the Sway (*y*) motion could cause turbulence because of the flow through the manifold, these motions are not taken into account in these experiments. For the experiments performed, the pole-placement algorithm in Section 4.2 was used to calculate the initial values for the FL controller gains which were finely tuned in situ once the robot was in the water. The theoretical values for the model parameters have been calculated using CAD software and the equations for hydrodynamic derivatives of the added mass force in (11), while omitting the linear and quadratic damping terms in (17).

Table 2, Table 3, Table 4 and Table 5 show the results of the transient state and steady-state time analysis evaluated on the response of both the PID and the FL control systems for each motion tested. The time response metrics chosen for the comparison of the performance were: overshoot percentage (OS%), rise time ($T_{r}$), fall time ($T_{f}$), settling time ($T_{s}$) and mean error in steady state (eSS). To evaluate the effectiveness of the FL controller developed in this work, the results obtained with the PID controller will be interpreted as the baseline for the desired controller responses. Thus, an effective control of the spherical UV with the FL controller will present lower errors in steady-state and faster transient times.

#### 5.2.1. Surge Motion

**Figure 9:**
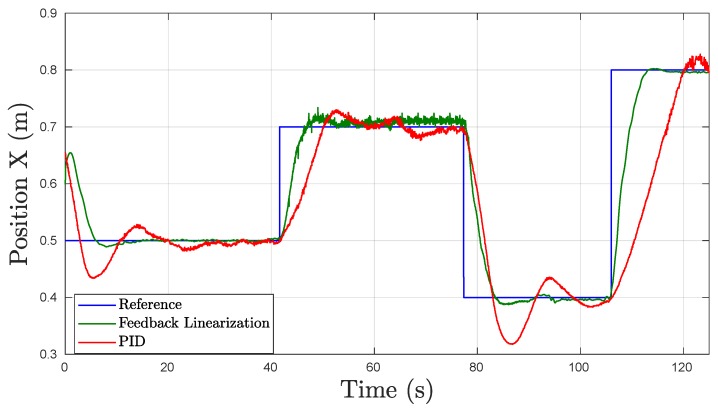
Experimental results obtained in underwater tests in Surge.

The experiments for the movement in surge were designed to validate the response of the UV to reference commands in the vehicles forward and backward directions of motion (*x* axis). Due to the limited FOV of the camera used to calculate the position feedback, the reference commands sent to the robot were constrained to a reduced range, thus, small steps were commanded. Figure 9 shows the position displacement in the *x* axis of the vehicle for the surge motion test. The UV was given an initial reference of 0.5 m which is movement in the forward direction towards the frame of the camera. Afterwards, multiple references are sent to verify the dynamics in each direction of movement.

As can be seen in Figure 9, the response of the FL controller is evidently more stable than that of the PID controller with clear overshoot by the PID controller for the set-points commanded at approximately t= 5 s and t= 85 s. Table 2 shows the comparison of the results obtained. During these tests, the FL controller was able to achieve less OS% while significantly reducing the $T_{r}$ from 6.83 s for the PID to 3.77 s and the $T_{s}$ from 24.94 s to 7.02 s. The PID controller averaged an eSS of 0.0083 m which is notable and sufficient for navigation purposes, while the FL controller obtained a lower eSS value of 0.0056 m. Nevertheless, during the FL controller tests, large fluctuations in the signal were detected from t= 40 s to t= 78 s which were a result of detection errors from the fiducial marker localization measurements due to turbidity in the experimental tank.

#### 5.2.2. Heave Motion

The depth hold performance is especially significant due to the working environment the UV will have. Therefore, high precision is needed to validate a controller as suitable to be tested in the real UNEXMIN mine tunnel exploring robot. The depth references sent to the UV were 10 cm steps to analyze the performance of the vehicle in maintaining precise depth positions.

**Figure 10:**
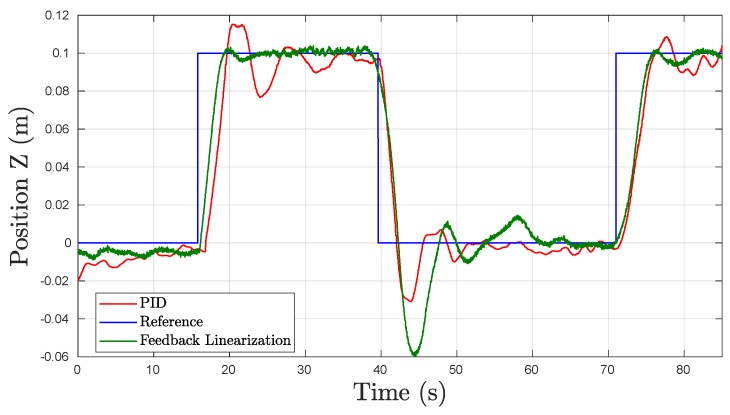
Experimental results obtained in underwater tests in Heave.

The dynamics of the heave tests, seen in Figure 10, showed very similar behaviors to the surge tests mentioned previously for both the PID and FL controllers. This was to be expected since the UV is symmetric in all three planes and thus must have similar dynamics in the linear motions. Both the PID controller and the FL controller achieved a similar $T_{r}$, while the FL controller once again outperformed the PID controller in terms of $T_{s}$ with values of 7.9 s and 17.8 s, respectively, and a considerable decrease in the eSS from 0.0063 m to 0.0020 m. Nevertheless, the PID controller obtained a lower OS% for the upward movement set-point at t= 45 s and a faster $T_{f}$ than the developed FL controller. This change in OS% could be due to differences in trimming the robot to be as neutrally buoyant as possible, thus changing the buoyant force and the behavior when surfacing.

#### 5.2.3. Yaw Motion

**Figure 11:**
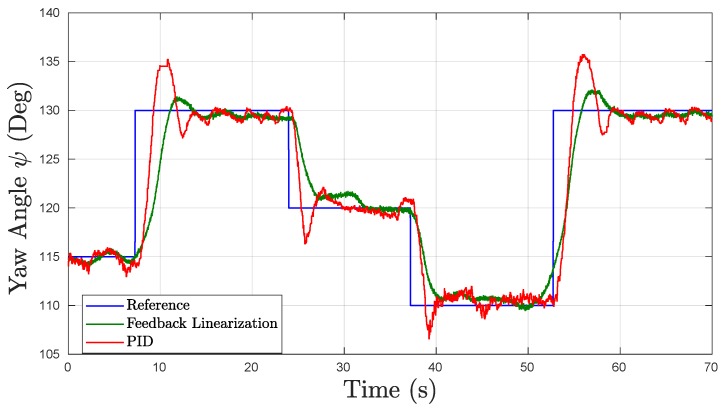
Experimental results obtained in underwater tests in Yaw.

Figure 11 presents the results for the yaw (ψ) underwater experimental tests. During this test, the depth was kept at a static set-point to examine the rotational dynamics individually. Even though the UV was equipped with an onboard autopilot which measures heading angles with an embedded magnetometer, the measurements obtained in initial testing were not useful and presented significant noise. Therefore, the visual marker pose estimation was used for yaw measurements as well. With the reduced size of the test tank (Figure 8), the FOV of the camera used for the fiducial marker localization allowed an interval of approximately [105°, 140°] for command references in yaw to validate the PID and FL controllers.

As can be seen in Table 4, the FL yaw controller provided an OS% of 1.07%, lower than the 4.24% achieved by the PID controller, as well as lower $T_{s}$ and eSS. The PID yaw controller, on the other hand, had a much faster $T_{r}$ and $T_{f}$. Nevertheless, as can be seen in Figure 11, the response for the PID controller showed undesirable oscillatory behavior in steady-state (SS) with a constant frequency and amplitude around the reference angle set at 130°. The response of the yaw controller should produce symmetric responses for both directions; however, the addition of the tether cable for data acquisition produced undesired drag in lateral direction which caused different control responses. Nonetheless, the controller compensated for these external disturbances and reached the reference angles.

#### 5.2.4. Pitch Motion

**Figure 12:**
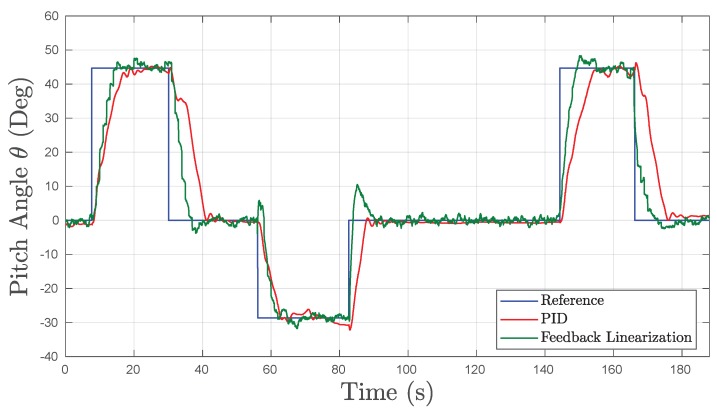
Experimental results obtained in underwater tests in Pitch.

As explained in Section 2 the pitch (θ) motion of the UV is achieved by the movement of an internal pendulum device which shifts the center of gravity. This movement in pitch allows the UV to navigate nose down (negative angles) or nose up (positive angles). The tests for the pitch motion had a duration of approximately 188 s. During this time, the UV was given steps in forward angle references of −30° (nose down) and backward references of +45° (nose up). The angle output of the UV to the reference step commands is shown in Figure 12.

The pitch angle of the UV is expected to be changed during normal operation in exploration tasks. One scenario is navigating nose down (θ=90°) in a vertical shaft and afterwards shifting the pitch to enter a horizontal tunnel. Therefore, fast transient response in pitch control is desired as well as a low error in steady state. As can be seen in Figure 12, both the PID and FL controllers were able to effectively control the pitch angle in transient and steady state. However, the performance of the FL controller exceeded that of the PID with faster rise and fall times of 5.11 s and 4.25 s, respectively, while obtaining a settling time 7.16 s faster and an error in steady-state of 0.9347°. Nonetheless, the PID controller achieved a substantially lower OS% of 1.28% compared to the 8.03% achieved by the FL controller. This difference could be due to the slower and less aggressive tuning of the PID controller where higher values tended to oscillate and become unstable.

## 6. Conclusions

In this paper, the design and control of a scaled prototype spherical underwater vehicle for flooded mine tunnel exploration was presented. The mechanical and electrical designs have been validated, as well as a novel pendulum mechanism for passive pitch control and a manifold thruster configuration for propulsion. The 6 DOF equations of motion of the underwater vehicle have been derived and simplified to reduced ordered models of longitudinal and lateral states. Surge, heave, pitch and yaw motions were tested in real underwater position hold experiments to evaluate the performance of the State FL controller and compare these to a baseline PID controller.

Results demonstrate that the spherical underwater vehicle was able to achieve all the required motions and that the State FL control method outperformed the classical control scheme in terms of lower overall errors and faster transient responses. Thus, The FL control algorithm tested experimentally in this work will be extended and implemented on the UX-1 robot for field testing in real underwater mine scenarios.

One of the unique features expected of this UV is the ability to stabilize in a completely nose-down (−90°) or nose-up (+90°) orientation which increases maneuverability and aids navigation tasks. Pitching the vehicle while navigating in the forward or backward direction, offers the possibility of adjusting to any slope found without the need for extra actuation of the UV heave motion. Due to the limitations of the scaled prototype used, in this work the 90 degrees pitch movement was not tested. Improvements will be made to achieve this range of pitch motion in future tests. Nevertheless, the pitch control results validate the pendulum mechanism design implemented in this work as an appropriate solution for the pitch stabilization and control of a spherical UV.

Future work on the development of this prototype can be divided in three areas: parameter identification, software, and hardware. To evaluate the effects of uncertainties in the model parameter calculations, used for these initial tests, experimental identification tests will be performed to obtain accurate model parameters to improve the controllers’ overall performance and explore parameter uncertainty adaptation limits with the current control method. The work to be performed on improving the hardware includes testing low frequency communication modules for wireless operation, testing the lateral system dynamics generated by the manifold system configuration, and obtaining the full expected motion of the pitch using the pendulum mechanism. The software to be implemented for future works includes the implementation and validation of additional advanced control techniques such as sliding mode control and $L_{1}$ Adaptive Control and sensor fusion algorithms to enhance the localization of the UV in underwater tests. Additionally, the redundancy in the use of 8 motors for propulsion will be tested by simulating motor failures during operation and evaluating the reliability of the design.

## Figures and Tables

**Table 1:** Actuator Configuration for Spherical UV.

Motion	Thruster Configuration
Surge	$\left\{T_{1},T_{5},-T_{3},-T_{7}\right\}$
Yaw	$\left\{T_{3},T_{5},-T_{1},-T_{7}\right\}$
Heave	$\left\{T_{4},T_{8},-T_{2},-T_{6}\right\}$
Roll	$\left\{T_{4},T_{6},-T_{2},-T_{8}\right\}$
Sway	$\left\{T_{1},T_{2},T_{3},T_{4},-T_{5},-T_{6},-T_{7},-T_{8}\right\}$

**Table 2:** Surge Time Response Results in Underwater Experiments for PID and FL Controllers.

Metrics	PID	FL	Units
OS%	13.16	11.98	[%]
$T_{r}$	6.83	3.77	[s]
$T_{f}$	3.17	2.18	[s]
$T_{s}$	24.94	7.02	[s]
eSS	0.0083	0.0056	[m]

**Table 3:** Heave Time Response Results in Underwater Experiments for PID and FL Controllers.

Metrics	PID	FL	Units
OS%	2.96	5.92	[%]
$T_{r}$	2.74	2.27	[s]
$T_{f}$	1.70	2.52	[s]
$T_{s}$	17.80	7.93	[s]
eSS	0.0063	0.0020	[m]

**Table 4:** Yaw Time Response Results in Underwater Experiments for PID and FL Controllers.

Metrics	PID	FL	Units
OS%	4.24	1.07	[%]
$T_{r}$	1.11	2.61	[s]
$T_{f}$	1.34	4.05	[s]
$T_{s}$	7.83	6.99	[s]
eSS	0.6522	0.6179	[°]

**Table 5:** Pitch Time Response Results in Underwater Experiments for PID and FL Controllers.

Metrics	PID	FL	Units
OS%	1.28	8.03	[%]
$T_{r}$	7.05	5.11	[s]
$T_{f}$	8.59	4.25	[s]
$T_{s}$	16.34	9.18	[s]
eSS	1.531	0.9347	[°]

## Abbreviations

The following abbreviations are used in this manuscript:

AUVs	Autonomous Underwater Vehicles
UV	Underwater Vehicle
COTS	Commercial off-the-shelf
CG	Center of Gravity
CB	Center of Buoyancy
DOF	Degrees of Freedom
ROS	Robot Operating System
SNAME	Society of Naval Architects and Marine Engineers
GPIO	General-Purpose Input/Output
ESC	Electronic Speed Controllers
NED	North-East-Down
FL	Feedback Linearization
PID	Proportional-Integral-Derivative
